# The imperative of evidence-based health workforce planning and implementation: lessons from nurses and midwives unemployment crisis in Ghana

**DOI:** 10.1186/s12960-020-0462-5

**Published:** 2020-03-06

**Authors:** James Avoka Asamani, Ninon P. Amertil, Hamza Ismaila, Francis Abande Akugri, Juliet Nabyonga-Orem

**Affiliations:** 1 World Health Organization, Regional Office for Africa, Inter-Country Support Team for Eastern and Southern Africa, Harare, Zimbabwe; 2 School of Nursing and Midwifery, Valley View University, Oyibi, Accra, Ghana; 3 Human Resources Division, Ghana Health Services, Accra, Ghana

**Keywords:** Human Resources for Health, Nursing and midwifery workforce, Nurse unemployment, Midwife unemployment, Health workforce policy

## Abstract

Following periods of health workforce crisis characterised by a severe shortage of nurses, midwives and doctors due to low production rates and excessive out-migration, the Government of Ghana through the Ministry of Health (MOH) responded by expanding training and allowing private sector involvement in the training of health workers especially nurses and midwives. This resulted in substantial increases in the production levels of nurses and midwives even above the projections of the MOH. In this paper, we discuss how a strategy that was seemingly well planned suffered a decade of uncorrected implementation lapses resulting in a lingering need-based shortage of nurses and midwives at service delivery points whilst thousands of trained nurses and midwives remained unemployed for up to 4 years and constantly protesting for jobs. In the short term, we argue that the Government of Ghana would need to increase investment to recruit trained and unemployed nurses and midwives whilst a comprehensive health labour market analysis is conducted to provide robust evidence towards the development of a long-term health workforce plan that would guide future production of nurses and midwives. The Government of Ghana may also explore the option of a managed migration programme to export nurses/midwives to countries that are already destinations to individual migration initiatives in a bid to mitigate the potential skill loss associated with long periods of unemployment after training, especially for those who trained from the private institutions.

## Introduction

Nurses and Midwives have played, and continue to play, critical roles in the health sector of Ghana. As part of efforts to address health workforce shortages, Ghana employed several strategies some of which have been applauded in one way or the other. Nevertheless, a seeming incoherence of, and non-adherence to, the stated policy of the Ministry of Health (MOH) has led to manifest unemployment of trained nurses and midwives due to limited fiscal space. The situation is beginning to threaten not only the quality and sustainability of nursing and midwifery education in Ghana but also the image and public confidence of the nursing and midwifery professions in the country. Based on our knowledge of the turn of events and backed with publicly available documents and media reportage, we examine the underlying policy implementation gaps and incoherence which spans over a decade.

## Background

Following periods of severe health workforce shortages in Ghana, the country was classified in the 2006 world health report by the World Health Organization (WHO) alongside others as experiencing a health workforce crisis [1]. The Government through the Ministry of Health (MOH) responded by embarking on a drive to expand and liberalise (allow profit-driven private sector participation) the training of health workers especially nurses and midwives [2]. This move together with the consolidation and improvement in the health sector salaries in 2006 [3], attracted overwhelming interest from qualified Ghanaians to join the health workforce, particularly the nursing and midwifery professions. Nursing and Midwifery Training Colleges (NMTCs) by 2007 started receiving large volumes of applicants beyond their admission capacities. Some of the applicants were said to have been attracted by the enhanced wages of nurses/midwives whilst others considered the nursing and midwifery professions as a springboard for other careers [3, 4] or an easy means to migrate abroad [5]. This translated into increased enrolment in the various nursing and midwifery training schools resulting in the production of nurses and midwives at levels that outstripped the Government’s projections at the time and the available fiscal capacity to absorb the new graduates. For instance, the nurse/midwife density increased from 4.5 per 10 000 population in 2006 to 21.2 per 10 000 population by 2016. The improved nursing and midwifery workforce numbers also contributed to the essential health workforce (doctors, nurses and midwives) density which also improved from 10.7 per 10 000 population in 2006 to 22 per 10 000 population by 2016. Despite these increases, the essential health workforce density was still below the international benchmark of 23 doctors, nurses and midwives per 10 000 population at the time [1]. Recent projections estimated that there is still 35% shortage of midwives, 33% for professional nurses and 6–11% for nursing assistants based on nationally determined staffing norms and standards [6]. When compared with the threshold of 45 health professionals per 10 000 population needed to make progress towards universal health coverage [7], the estimated shortage of nurses and midwives could even be higher. Nevertheless, the substantial increase in the health workforce particularly nurses and midwives concomitantly impacted on the public health sector wage bill which has ballooned from GH¢ 373 million (US$ 77.7 million) in 2010 to GH¢ 1.9 billion (US$ 394.7 million) in 2017 representing 408% increase—at least 40% annual wage bill increase after adjusting for inflation [8, 9]. Since 2011, the wage bill alone has been said to be constituting 80–95% of the total government’s budgetary allocation to the Ministry of Health (excluding budget for the National Health Insurance Scheme) [10].

Whilst there are several angles to the Ghanaian health workforce issues from a labour market and policy perspectives, in this piece, we focus mainly on policy implementation lapses which we contend partly (if not greatly) contributed to the lingering ‘paradoxical unemployment’ (existence of unemployment despite a critical need for the services of the unemployed) of nurses and midwives.

### From policy implementation gaps to a policy vacuum

**Fig. 1:**
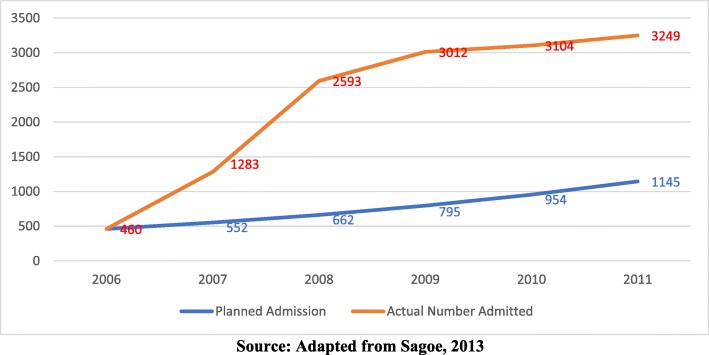
Trend of planned and actual admissions for enrolled nurses in Ghana, 2006–2011 (source: adapted from Sagoe, 2013)

On the back of the aforesaid increased volume of nursing and midwifery applicants, admissions into the NMTCs were further expanded above the stated training plan of the MOH which was contained in the Human Resources for Health (HRH) policy and strategy, 2007–2011 [11]. For example, Sagoe [11] reported that instead of a planned average annual increase of 20% in the training of enrolled nurses from 2006 to 2011, there was an annual average increase of 61% without wage bill impact analysis or economic feasibility analysis for their employment when they pass out and also without regard to the impact of the large increases on the quality of training (see Fig. 1).

The expansion of nursing and midwifery training was further escalated without conducting or updating the needs assessment (need-based forecast) after the expiration of the strategic plan and its projections in 2011. From that time, there was no more an officially approved strategic plan to guide the health workforce production—a draft was developed for the period 2012–2016 but never got approved by the National Development Planning Commission (NDPC) as of the end of 2018. During this period of a substantive policy vacuum, heads of NMTCs focused on the income generation side of large admissions and often negotiated for higher admission quotas without recourse to employment absorption capacity or need. Meanwhile, from 2015, the fiscal space for increasing public health sector wage bill declined as part of larger governmental fiscal control measures [12–14]—thereby constraining absorption capacity for new health graduates including nurses and midwives.

### ‘Knee-jerk’ policy reactions

**Fig. 2:**
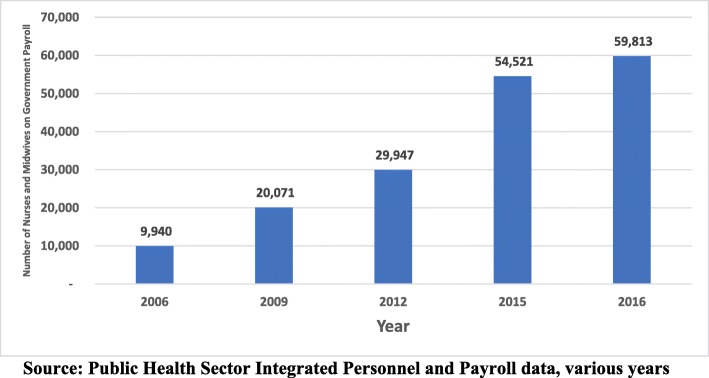
Growth trend in nursing and midwifery in the public sector of Ghana (source: Public Health Sector Integrated Personnel and Payroll data, various years)

Without a documented health workforce development policy or strategic plan, between 2012 and 2016, the stock of the nursing and midwifery workforce in the public sector alone increased at a rate of about 25% annually. Overall, the public sector nurses and midwives increased by more than 500% from 9940 in 2006 to 59 813 in 2016 (see Fig. 2). This was in the context of the number of institutions offering nursing programmes continuing to increase in both numbers and size—some of which have been described as sub-standard in terms of infrastructure, equipment and faculty [15, 16]. These increases coupled with the constrained fiscal space, in the absence of a substantive plan, saw some ad hoc decisions or knee-jerk reactions to deal with issues concerning the overall health workforce planning, development and recruitment.

First, enrolled nurses and community health nurses who recorded the most increases in the rate of production had limited career pathways at the time and agitated for better recognition and enhanced career advancement opportunities [17]. The situation ignited a discourse about the sustainability of the rate of production of these cadres of nurses. The ensuing tension necessitated the creation of an ‘escape valve’—the introduction of top-up programmes where enrolled nurses and community health nurses could become professional nurses or midwives through bridging courses for diplomas and degrees that are offered by virtually all the universities in the country. This also led to an unintended consequence where the academic institutions (both public and private) have set themselves in a competition to generate more revenues from the community health nurses and enrolled nurses by admitting large numbers in classes, potentially compromising the quality of training and engendering a situation the country could be getting ill-prepared nurses and midwives to further complicate a huge public outcry about a falling standard and quality of nursing and midwifery care.

Faced with financial constraints to recruit all the trained nurses and midwives [18], another knee-jerk reaction was to restrict the recruitment of nurses and midwives to only those that trained in public institutions, who were provided Government’s stipend and bonded to serve the country upon completion, to the neglect of those that trained in private schools and universities [19]. This decision attracted considerable public debate and contestation about its fairness to nurses/midwives trained at the private institutions using their resources and without benefitting from government stipend but denied public sector employment. This resulted in the formation of various interest groups of nurses and midwives seeking employment who picket occasionally at government installations to advocate for jobs [20–22]. Between 2016 and 2018, public protest and picketing by nurses and midwives demanding for jobs became (and continue to be) routine and arguably took centre stage in national policy and political discourse.

In 2018, for example, it was estimated that nearly 22 000 nurses and midwives were completing training to be ready for employment by 2019 which would add GH¢ 3151.3 million (US$ 606 million) annually to the wage bill of an already overstretched budget of the MOH (see Table 1). Although the private health sector in Ghana is expanding, it employs less than a tenth of the nurses and midwives produced [23]; hence, most nurse/midwife graduates seek to be employed mainly in the publicly funded health facilities. However, under an extended credit facility agreement with the International Monetary Fund (IMF) with attendant austerity measures, all those who graduated from the public NMTCs between 2016 and 2018 could not be immediately employed (in addition to those who attended private institutions and qualified from 2015)^1^. Reportedly, MOH at the time estimated that the total number of unemployed nurses and midwives as of January 2019 were over 40 000 [21, 24]—some of who have not been included in the estimates that are reflected in Table 1. Even though the Government has promised to employ all of them [24], another concern that appears to be overlooked is the possible skills loss that might have occurred during the long waiting period (of unemployment) which could compromise the quality of care if these nurses and midwives are employed without appropriate mentorship and/or re-training.

**Table 1:** Expected output from Nursing and Midwifery Training Colleges, 2017—2020

Duration of training	Category of trainees	Number to be ready for employment				Cost estimates (in million Ghana cedis)—additional wage bill				Cost estimates (in million US dollars)—additional wage bill			
		2017	2018	2019	2020	2017	2018	2019	2020	2017	2018	2019	2020
2-year programmes	Community health nursing	3 922	4 218	N/A	N/A	437.7	470.6	N/A	N/A	84.2	90.5	N/A	N/A
	Enrolled nursing	6 142	6 426	N/A	N/A	685.3	717.0	N/A	N/A	131.8	137.9	N/A	N/A
	Post-basic midwifery	765	854	N/A	N/A	132.3	147.8	N/A	N/A	25.4	28.4	N/A	N/A
3- or 4-year programmes	Registered community health nursing	384	922	1,290	643	66.4	159.4	223.2	111.3	12.8	30.7	42.9	21.4
	Registered general nursing	3 630	6 427	6 412	5 627	627.8	1 111.7	1 109.0	973.3	120.7	213.8	213.3	187.2
	Registered mental health nursing	351	388	1 881	1 924	60.7	67.1	325.3	332.8	11.7	12.9	62.6	64.0
	Registered midwifery	1 598	2 762	3 139	3 007	276.3	477.8	543.0	520.1	53.1	91.9	104.4	100.0
Total		**16 792**	**21 997**	**12 722**	**11 201**	**2 286.5**	**3 151.4**	**2 200.5**	**1 937.4**	**439.7**	**606.0**	**423.2**	**372.6**

Source: presentation to the Health Sector Working Group of the MOH—June 2017; data on expected graduates taken from the Nursing and Midwifery Council and salary data taken from MOH. Notes: (1) the numbers relate to the expected outputs only and do not account for existing trained but unemployed nurses/midwives; (2) estimates are based on 80% pass rate from the NMC; (3) N/A is indicated for 2-year programmes against 2019 and 2020 because admissions were yet to be done at the time of the analysis; (4) estimates for 2019 and 2020 are lower than previous years because of admissions into the 2-year programmes had not been done and, thus, not taken into consideration; (5) an exchange rate of US$ 1 to GH¢ 5.2 used based on the average exchange rate for the first quarter of 2019. The costs are adjusted for inflation assumed at the rate of 10% based on the trend of recent years.

### Suggestions for policy consideration

#### Streamlining nursing and midwifery production

From 2017, MOH in collaboration with the Nursing and Midwifery Council (NMC) attempted to streamline the training of nurses and midwives by giving NMTCs admission quotas which were justified to Members of Parliament as evidence-based [25]. However, the implementation is said to be weak, and the quotas largely ignored by heads of the NMTCs especially from 2018 mainly due to the income generation considerations and persistent high demand for admissions into the NMTCs. It may be worth opening a discussion on reviewing the admission criteria into various nursing and midwifery programmes alongside a stricter implementation of the admission quotas which was instituted in 2017.

### Generate robust health labour market evidence for health workforce policies and plans

In developing a new HRH policy and strategy, it would be imperative for the MOH to undertake a comprehensive health labour market analysis with particular attention paid to the nursing and midwifery labour market dynamics in generating robust evidence to support need-based training of nurses and midwives which will not only meet population health needs but also be balanced with quality of training and economic capacity for absorption. In this regard, the need for broader stakeholder engagement to validate the results of the health labour market analysis leading to the development of the HRH policy/strategy cannot be overemphasised.

### Stimulate private sector employment and dual practice regulation

Although the private-for-profit health sector constitutes about 20% of health service delivery in Ghana, they have for a long time been employing less than 10% of the health workforce on a full-time basis. Instead, the private-for-profit health sector has historically relied on the dual practice of public sector employees [23]. Given this inertia of the private-for-profit health sector in expanding health worker employment that will contribute to clear the backlog of unemployed nurses and midwives, it would be imperative for the government to include stricter fulltime employment staffing standards for the accreditation of private health facilities alongside employment-conditioned tax incentives to stimulate health sector job creation.

### Improve government investment in health sector job creation

There are several ongoing health infrastructure projects (although some remained uncompleted for many years) which when completed would require health workers including nurses and midwives to function. As part of the aspiration towards universal health coverage, the government needs to increase its investment in health especially in the areas of infrastructure and employment to absorb those who are already qualified but unemployed to fill the existing and future staffing gaps in health facilities and communities. Admittedly, however, the government within its constrained resources may be unable to employ all the nurses and midwives who are expected to qualify from both public and private institutions in the coming years if the mix and rate of production are not rationalised based on the robust evidence from a health labour market analysis. An attempt to do so would only be cosmetic and unsustainable. Government health facilities could be empowered to apply a portion of their internally generated funds to the recruitment of health workers to fill critical shortages. This, however, must be linked to improving on the erratic reimbursement for services from the National Health Insurance Scheme which represents the bulk of income for health facilities.

### Institute a managed migration programme

Already, the nurse/midwife unemployment situation is said to be fuelling out-migration to high-income countries where there is a demand-related shortage of nurses and midwives [26, 27], a situation that has a potential of reigniting the mass brain drain that occurred in Ghana in the late 1990s. With this reality, it may be necessary for the medium term for the Government through the MOH to begin exploring opportunities for ‘managed migration’ of nurses and midwives by exporting nursing and midwifery skills to other countries that are in need and can afford. This is not because Ghana necessarily has more nurses and midwives than needed, but mainly because it cannot financially afford to absorb all those that have been trained or are expected to be trained. Such a move could mitigate the potential skill loss associated with the 2 to 4 years waiting period after training before employment especially for those who trained from the private institutions. With this, the intent could be that the nurses and midwives who are sent to other countries under the ‘managed migration programme’ would compulsorily return after a specified period, hopefully with added value in terms of enhanced skills and experience to also support the Ghanaian healthcare system.

## Conclusion

In an attempt to address a severe shortage of nurses and midwives, Ghana expanded and liberalised their training which alongside uncorrected policy implementation lapses resulted in training outputs outstripping economic capacity for absorption. Therefore, whilst there is a lingering need-based shortage of nurses and midwives, thousands remain unemployed for 2–4 years which is adversely impacting on the skills they acquired in training.

The lessons from Ghana’s experience has shown that in scaling up the production of the health workforce, it is not only essential to have an elaborate national plan informed by a comprehensive health labour market assessment, but it is also imperative to effectively monitor its implementation with the view of making the necessary adjustments. In liberalising the training of health workers, a strong regulatory mechanism is a prerequisite to ensure quality and alignment with national targets.

## Data Availability

The datasets supporting our conclusions are publicly available and will be provided upon request.

## Abbreviations

HWF: Health workforce
SDGs: Sustainable development goals
UHC: Universal health coverage
WHO: World Health Organization
NMTC: Nursing and Midwifery Training College
HRH: Human Resource for Health
MOH: Ministry of Health

## Footnotes

1 It is important to emphasise that there is so far no established evidence of oversupply of professional nurses and midwives based on population health needs in Ghana, but what is clear is a significant difficulty in creating economic demand for absorbing those trained.

## References

[1] WHO. The World Health Report: 2006: working together for health. 2006 [cited 2016 Oct 10]; Available from: http://apps.who.int/iris/handle/10665/43432.

[2] MOH. Human resource policies and strategies for the health sector, 2007-2011 [Internet]. Ministry of Health, Ghana; 2007. Available from: www.moh.gov.gh.

[3] Antwi J, Phillips DC. Wages and health worker retention: evidence from public sector wage reforms in Ghana. J Dev Econ [Internet]. 2013 [cited 2017 Jan 17];102:101–115. Available from: http://www.sciencedirect.com/science/article/pii/S0304387812000880.

[4] Abugri A, Jarvis M-A (2018). Northern Ghana final-year nurses’ attitudes towards nursing and remaining post qualification. Curationis..

[5] Abuosi Aaron Asibi, Abor Patience Aseweh (2014). Migration Intentions of Nursing Students in Ghana: Implications for Human Resource Development in the Health Sector. Journal of International Migration and Integration.

[6] Asamani JA, Chebere MM, Barton PM, D’Almeida SA, Odame EA, Oppong R (2018). Forecast of healthcare facilities and health workforce requirements for the public sector in Ghana, 2016–2026. Int J Health Policy Manag..

[7] WHO WHO (2016). Global Strategy on Human Resources for Health: workforce 2030.

[8] GHS. Human Resource Annual Report - 2017. Accra: Ghana Health Service; 2017.

[9] GHS. Health workforce gaps and cost-benefit analysis of proposed staff redistribution in the Ghana Health Service. Accra: Ghana Health Service; 2018.

[10] MOH. Health sector holistic assessment of the health sector performance - 2017. Ministry of Health, Ghana; 2017.

[11] Sagoe K. Human Resources for Health - challenges and strategies to address them in Sub-Saharan Africa. In 2013 [cited 2019 Mar 24]. Available from: http://www.fatoafrique.org/congres2013/IMG/pdf/sagoe_kenneth_ppt_fr.pdf.

[12] IMF. Press release: IMF approves US$918 million ECF arrangement to help Ghana boost growth, jobs and stability [Internet]. 2015 [cited 2016 Aug 11]. Available from: https://www.imf.org/en/News/Articles/2015/09/14/01/49/pr15159.

[13] MOF. The budget statement and economic policy of the Government of Ghana for the 2016 financial year presented to parliament [Internet]. Government of Ghana; 2015 [cited 2016 May 20]. Available from: www.mofep.gov.gh.

[14] MOF. End-year report on the budget statement and economic policy of the Republic of Ghana for the 2015 financial year [Internet]. Government of Ghana; 2016 [cited 2016 May 20]. Available from: www.mofep.gov.gh.

[15] Bell Sue Anne, Rominski Sarah, Bam Victoria, Donkor Ernestina, Lori Jody (2013). Analysis of nursing education in Ghana: Priorities for scaling-up the nursing workforce. Nursing & Health Sciences.

[16] Donkor ES. Nursing education in Ghana: an insight into undergraduate and graduate programmes. Footpr Nurs Prof Curr Trends Emerg Issues Ghana [Internet]. 2014 [cited 2014 Aug 13]; Available from: http://books.google.com/books?hl=en&lr=&id=BTkKBAAAQBAJ&oi=fnd&pg=PA1&dq=nursing+education+in+ghana&ots=HiYRoWgK0X&sig=QAs2wOUGdJx8R90IjAeg-iK-uDk.

[17] Modern Ghana. Community health nurses embark on indefinite strike [Internet]. Modern Ghana. 2016 [cited 2019 Apr 4]. Available from: https://www.modernghana.com/news/695617/community-health-nurses-embark-on-indefinite-strike.html.

[18] Peace FM Online. 2,000 nurses, midwives jobless -Govt has no money to employ them [Internet]. 2015 [cited 2019 Apr 4]. Available from: http://www.peacefmonline.com/pages/local/news/201507/247073.php.

[19] Adogla-Bessa D. Gov’t discriminating against private nurses – coalition | [Internet]. 2016 [cited 2019 Apr 4]. Available from: http://citifmonline.com/2016/05/09/govt-discriminating-against-private-nurses-coalition/.

[20] Andoh D. Unemployed private nurses to demonstrate [Internet]. Ghana Trade - Official SME Product Portal and Web Gallery. 2017 [cited 2019 Mar 24]. Available from: http://ghanatrade.com.gh/Latest-News/unemployed-private-nurses-to-demonstrate.html.

[21] Ansah M. Unemployed graduate nurses protest against gov’t in Kumasi [Internet]. Citi Newsroom. 2018 [cited 2019 May 1]. Available from: https://citinewsroom.com/2018/09/05/unemployed-graduate-nurses-protest-against-govt-in-kumasi/.

[22] Elorm. Bonded nurses and midwives demand postings to health facilities [Internet]. Ghana Business News. 2018 [cited 2019 Mar 24]. Available from: https://www.ghanabusinessnews.com/2018/09/07/bonded-nurses-and-midwives-demand-postings-to-health-facilities/.

[23] Saleh K. The health sector in Ghana: a comprehensive assessment: World Bank Publications; 2013.

[24] Abedu-Kennedy D. Government to recruit 40,000 nurses in 2019 - Health Minister [Internet]. AdomOnline.com. 2018 [cited 2019 Mar 24]. Available from: https://www.adomonline.com/ghana-news/government-to-recruit-40000-nurses-in-2019-health-minister/.

[25] Parliament of Ghana. Hansard - official report of parliamentary debates [Internet]. Accra; 2017 Oct [cited 2019 Apr 5]. Report No.: 99. Available from: https://www.parliament.gh/epanel/docs/pb/6th%20October,%2020.pdf#viewer.action = download.

[26] Scheffler RM, Campbell J, Cometto G, Maeda A, Liu J, Bruckner TA (2018). Forecasting imbalances in the global health labor market and devising policy responses. Hum Resour Health..

[27] Asamani JA, Nabyonga-Orem J. Will the 2019 UK Election impact on the health workforce in Africa? Feels like a rhetorical question… [Internet]. International Health Policies. 2019 [cited 2019 Dec 19]. Available from: https://www.internationalhealthpolicies.org/blogs/will-the-2019-uk-election-impact-on-the-health-workforce-in-africa-feels-like-a-rhetorical-question/.

